# Prevalence and functional profile of SARS-CoV-2 T cells in asymptomatic Kenyan adults

**DOI:** 10.1172/JCI170011

**Published:** 2023-07-03

**Authors:** Taraz Samandari, Joshua B. Ongalo, Kimberly D. McCarthy, Richard K. Biegon, Philister A. Madiega, Anne Mithika, Joseph Orinda, Grace M. Mboya, Patrick Mwaura, Omu Anzala, Clayton Onyango, Fredrick O. Oluoch, Eric Osoro, Charles-Antoine Dutertre, Nicole Tan, Shou Kit Hang, Smrithi Hariharaputran, David C. Lye, Amy Herman-Roloff, Nina Le Bert, Antonio Bertoletti

**Affiliations:** 1 US Centers for Disease Control and Prevention, Nairobi, Kenya.; 2 Kenya Medical Research Institute (KEMRI), Centre for Global Health Research, Kisumu, Kenya.; 3 Moi University School of Medicine, Immunology Section, Eldoret, Kenya.; 4 KAVI Institute of Research, University of Nairobi, Nairobi, Kenya.; 5 County Government of Kisumu, Department of Health and Sanitation, Kisumu, Kenya.; 6 Washington State University Global Health Kenya, Nairobi, Kenya.; 7 Paul G. Allen School of Global Health, Washington State University, Pullman, Washington, USA.; 8 Gustave Roussy Cancer Campus, Villejuif, France.; 9 Institut National de la Santé et de la Recherche Médicale (INSERM) U1015, Equipe Labellisée — Ligue Nationale contre le Cancer, Villejuif, France.; 10 Programme in Emerging Infectious Diseases, Duke University–National University of Singapore Medical School, Singapore.; 11 National Centre for Infectious Diseases, Singapore.; 12 Tan Tock Seng Hospital, Singapore.; 13 Yong Loo Lin School of Medicine, National University of Singapore, Singapore.; 14 Lee Kong Chian School of Medicine, Nanyang Technological University, Singapore.; 15 Singapore Immunology Network, Agency for Science, Technology and Research (A*STAR), Singapore.

**Keywords:** COVID-19, Infectious disease, T cells

## Abstract

**Background:**

SARS-CoV-2 infection in Africa has been characterized by a less severe disease profile than what has been observed elsewhere, but the profile of SARS-CoV-2–specific adaptive immunity in these mainly asymptomatic patients has not, to our knowledge, been analyzed.

**Methods:**

We collected blood samples from residents of rural Kenya (*n* = 80), who had not experienced any respiratory symptoms or had contact with individuals with COVID-19 and had not received COVID-19 vaccines. We analyzed spike-specific antibodies and T cells specific for SARS-CoV-2 structural (membrane, nucleocapsid, and spike) and accessory (ORF3a, ORF7, ORF8) proteins. Pre-pandemic blood samples collected in Nairobi (*n* = 13) and blood samples from mild-to-moderately symptomatic COVID-19 convalescent patients (*n* = 36) living in the urban environment of Singapore were also studied.

**Results:**

Among asymptomatic Africans, we detected anti-spike antibodies in 41.0% of the samples and T cell responses against 2 or more SARS-CoV-2 proteins in 82.5% of samples examined. Such a pattern was absent in the pre-pandemic samples. Furthermore, distinct from cellular immunity in European and Asian COVID-19 convalescents, we observed strong T cell immunogenicity against viral accessory proteins (ORF3a, ORF8) but not structural proteins, as well as a higher IL-10/IFN-γ cytokine ratio profile.

**Conclusions:**

The high incidence of T cell responses against different SARS-CoV-2 proteins in seronegative participants suggests that serosurveys underestimate SARS-CoV-2 prevalence in settings where asymptomatic infections prevail. The functional and antigen-specific profile of SARS-CoV-2–specific T cells in African individuals suggests that environmental factors can play a role in the development of protective antiviral immunity.

**Funding:**

US Centers for Disease Control and Prevention, Division of Global Health Protection; the Singapore Ministry of Health’s National Medical Research Council (COVID19RF3-0060, COVID19RF-001, COVID19RF-008, MOH-StaR17Nov-0001).

## Graphical abstract

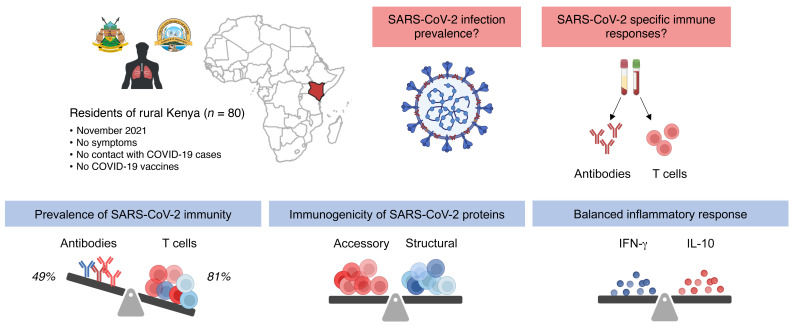

## Introduction

SARS-CoV-2 infection in Africa is characterized by a low number of mild and severe cases of disease (1, 2). The incidence of severe COVID-19 has been particularly low in Kenya (3, 4). Even though an undercount of deaths from COVID-19 cannot be firmly excluded, Kenya’s National Emergency Operations Centre reported that approximately 90% of infected individuals were asymptomatic during the COVID-19 pandemic (3, 4). This might be mainly explained by the country’s youthful population (i.e., median of 19 years for Kenya versus 38 for the United States), but other factors such as cross-reactive immunity induced by other coronaviruses (5) or commensal microorganisms (6), trained immunity stimulated by live vaccines (i.e., bacille Calmette-Guérin [BCG] and live oral polio vaccines) (7, 8), or a downregulation of the inflammatory response via helminth coinfection (9) could play mitigating roles.

The prevalence of past infection is classically measured using serological assays and in Kenya, by January–March 2021, blood donor anti-spike antibody seroprevalence ranged from 38% in rural western counties to 62% in the capital city, Nairobi (10). However, it has been repeatedly shown that among asymptomatic SARS-CoV-2–infected individuals, antibody levels are frequently low or absent, while T cell responses remain detectable (11–13). Furthermore, kinetics studies showed that memory T cells (14) persist longer than antibodies (15) in the blood, implying that seroprevalence as an indicator may underestimate the extent of asymptomatic SARS-CoV-2 exposure.

Here, we studied SARS-CoV-2–specific humoral and cellular immune responses in individuals from Kenya who never reported any symptoms of respiratory infection and who were not knowingly in contact with patients with COVID-19. We enlisted the guidance of locally-resident community health care workers to identify study participants residing in rural areas of the counties Kisumu and Elgeyo Marakwet, 2 regions of Kenya that by December 7, 2021, had reported 569 and 94 cases of COVID-19 per 100,000 population, respectively. Samples collected during November and December 2021 were studied in parallel for the presence of spike-specific antibodies and for T cells specific for SARS-CoV-2 structural (membrane, nucleoprotein [NP], spike) and accessory (ORF3a, ORF7, ORF8) proteins utilizing different methods of T cell characterization. Until now, SARS-CoV-2–specific T cells, which have been hypothesized to play a major role in the control of disease severity (16), have only been examined in African populations with convalescent COVID-19 (17, 18), while T cell response characteristics in asymptomatic Africans have never been studied.

## Results

**Figure 1:**
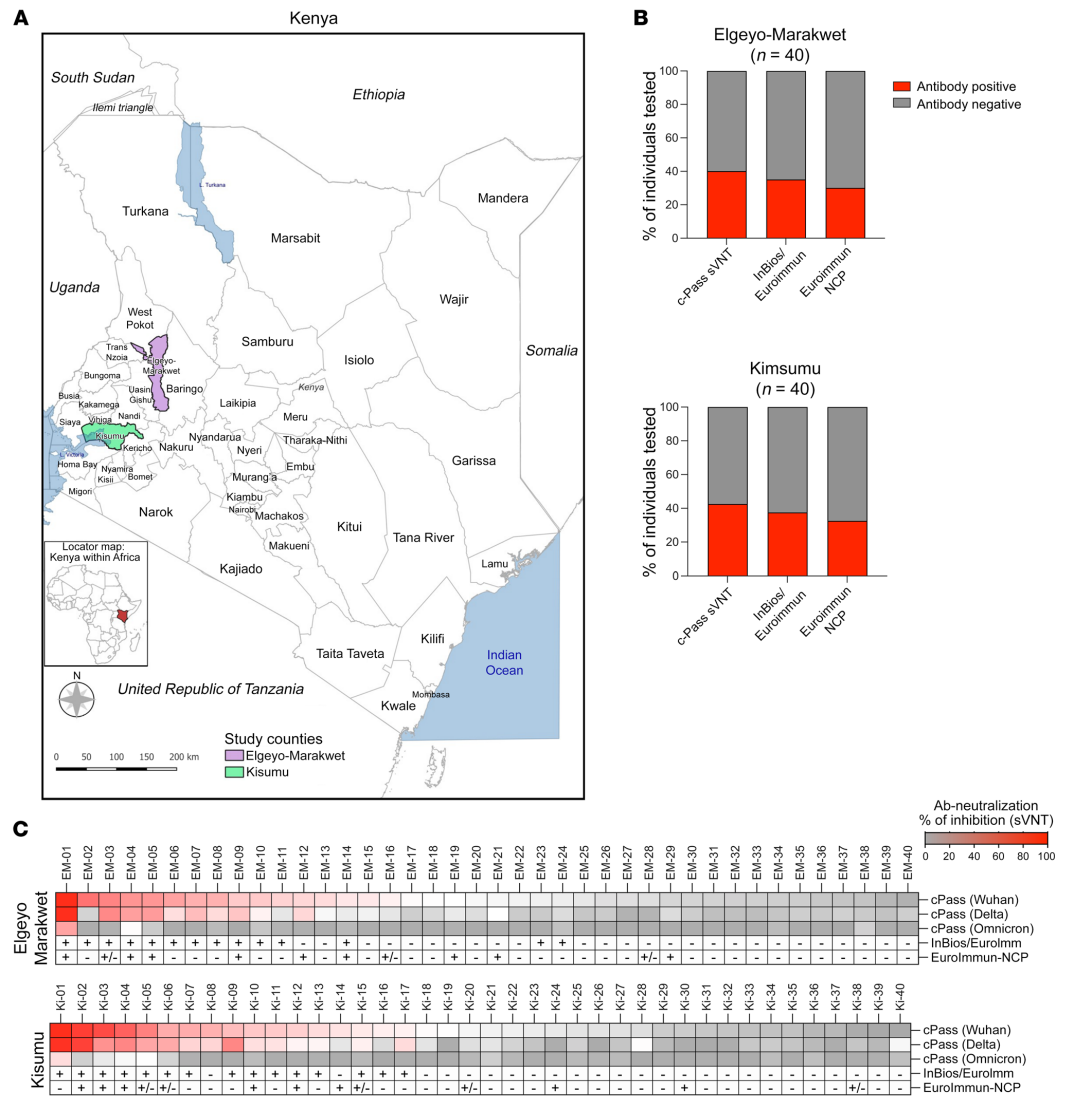
SARS-CoV-2 seropositivity in asymptomatic individuals living in Elgeyo Marakwet and Kisumu. (**A**) Individuals with no history of COVID-19 symptoms (including cough, shortness of breath, fever, or sinus congestion) and no contact with confirmed SARS-CoV-2–infected individuals were recruited in 2 counties of Kenya highlighted in purple and green. Elgeyo Marakwet (*n* = 40 participants); Kisumu (*n* = 40 participants). (**B**) Sera from participants were analyzed with 3 antibody tests measuring spike-specific antibodies: cPass measuring SARS-CoV-2–neutralizing antibodies with a surrogate virus neutralization test (sVNT); InBios SCoV-2 Detect IgG ELISA as an initial test followed by the EUROIMMUN anti–SARS-CoV-2 test as a confirmatory test for participants who were positive by InBios; and 1 antibody test, EUROIMMUN-NCP, measuring NP-specific antibodies. The percentages of participants positive for the different tests are shown in red. (**C**) Antibody data are shown for the individual participants from Elgeyo Marakwet and Kisumu. All participants were tested with the cPass sVNT against the spike protein of the first SARS-CoV-2 variant (Wuhan-Hu-1 strain) and the spike protein of the SARS-CoV-2 variants Delta and Omicron. In addition, test results from the InBios/EUROIMMUN and EUROIMMUN-NCP tests are shown (+, positive; –, negative; ±, borderline).

Study participants with no history of respiratory illness (cough, shortness of breath, fever, or sinus congestion) since December 2019, no contact with individuals known to have COVID-19, and no history of COVID-19 vaccination were recruited from Elgeyo Marakwet (*n* = 40) and Kisumu (*n* = 40; Figure 1A) counties. Among the participants, 42 of 80 (53%) were female and 65 of 80 (81%) were under the age of 50 years; 10 of 63 (16%) were HIV infected, the remainder (*n* = 17) having declined HIV testing, and 5 of 80 (6%) had hypertension (Table 1). All had negative nasal swab tests for SARS-CoV-2 by PCR. Antibodies specific for spike antigens were first tested using a surrogate neutralizing antibody test (GenScript cPass) (19) as well as anti–IgG-SARS-CoV-2 tests (InBios followed by EUROIMMUN assay; see Methods) measuring the level of IgG against the S1 region of the spike protein. We also tested NP-specific antibodies (anti–SARS-CoV-2 NCP ELISA, EUROIMMUN). Surrogate neutralizing antibodies were detected in 16 of 40 (40%) participants from Elgeyo Marakwet and in 18 of 40 (43%) participants from Kisumu (Figure 1B). Similar proportions were observed using the alternative InBios-EUROIMMUN assays (Figure 1B). To exclude the possibility that a primary infection with SARS-CoV-2 variants of concern (Delta, Omicron) might have induced spike-specific antibodies that do not cross-react with the Wuhan-Hu-1 spike protein, we tested for the presence of antibodies specific for the receptor-binding domain (RBD) region of Delta and Omicron. There was an almost complete concordance between the detection of antibodies against Wuhan-Hu-1 and Delta spike RBDs (Figure 1C). Only 2 individuals had antibodies specific for the Delta spike RBD in the absence of antibodies against the Wuhan spike RBD, while antibodies against the Omicron spike RBD were absent in all individuals except the 2 who had the higher values of pseudoneutralizing activity. This observation is consistent with evidence that the Delta SARS-CoV-2 variant was circulating in Kenya in the second quarter of 2021, while Omicron started to be detected in Kenya only in late November 2021 (20).

**Figure 2:**
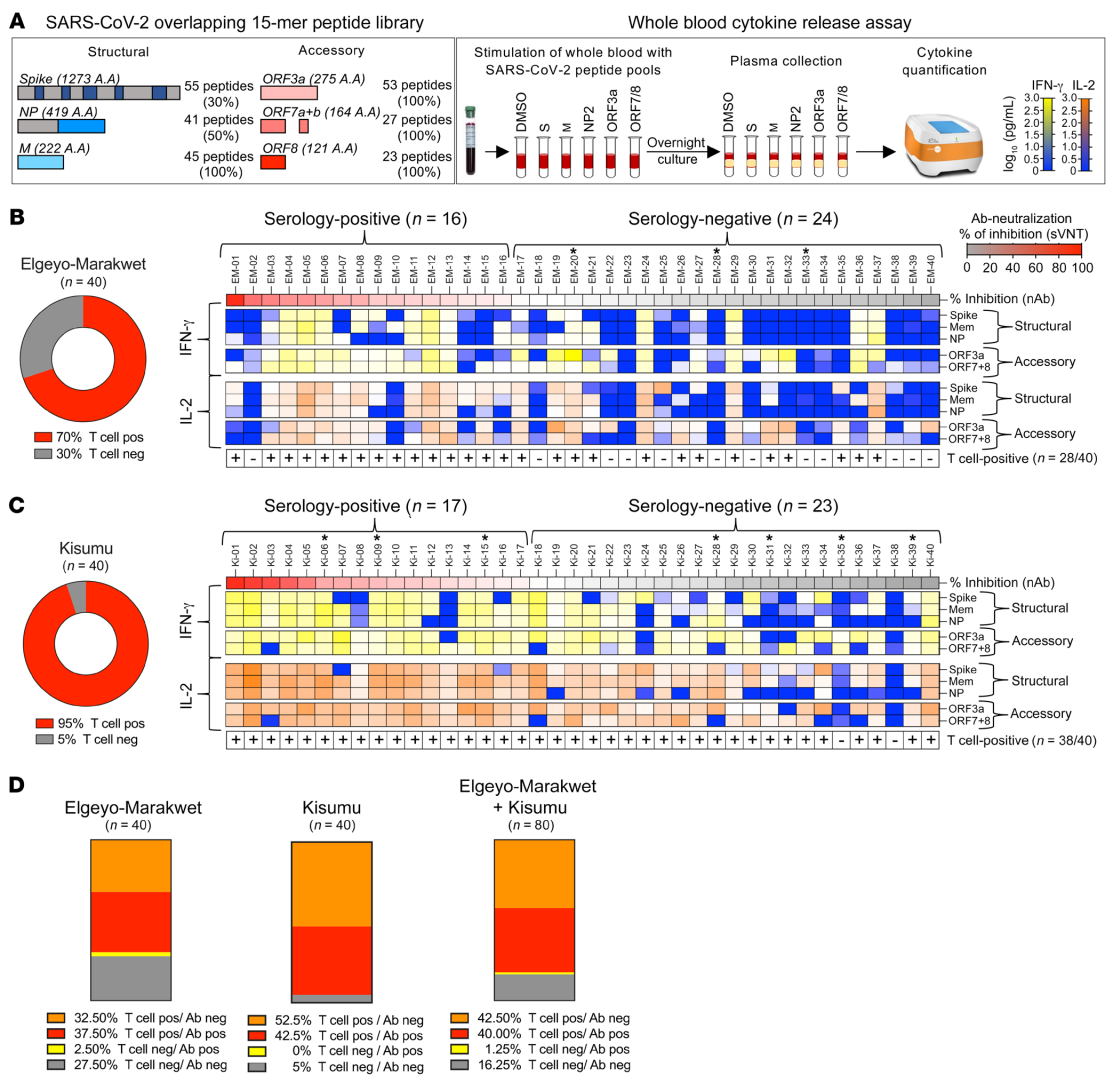
T cell response specific for different SARS-CoV-2 proteins in asymptomatic participants from Elgeyo Marakwet and Kisumu. (**A**) Schematic representation of the 5 SARS-CoV-2–specific peptide pools containing 15 mer overlapping peptides spanning 30% of spike (S), entire membrane (M), and 50% NP, and complete accessory proteins ORF3a and ORF7/8 that were used in 18-hour whole-blood cultures. Levels of IFN-γ and IL-2 secreted in response to peptide stimulation were quantified in the plasma of the blood cultures. If peptide stimulation induced greater than 5 pg/mL cytokines (IFN-γ and/or IL-2) above the corresponding DMSO controls with 2 distinct peptide pools, the individual was considered positive for SARS-CoV-2–specific T cells. The percentages of individuals from (**B**) Elgeyo Marakwet and (**C**) Kisumu who were positive for SARS-CoV-2–specific T cells are shown in red. Heatmaps show the levels of cytokines (yellow to blue: IFN-γ; orange to blue: IL-2) released upon stimulation with the distinct peptide pools in each individual; HIV-positive individuals are indicated by a single asterisk (*); samples considered positive for T cells are indicated by a plus sign (+). Participants’ data are organized according to the level of neutralizing antibodies. (**D**) Percentage of individuals who tested positive or negative for SARS-CoV-2–specific T cells and neutralizing antibodies. Mem, membrane; ORF7+8, ORF7 and ORF8; pos, positive; neg, negative.

We tested T cell reactivity against SARS-CoV-2 structural (spike, NP, membrane) and accessory (ORF3a, ORF7a/b, ORF8) proteins by stimulating whole blood within 8 hours from sample collection using 5 distinct peptide pools (Figure 2A, left). After overnight incubation, IFN-γ and IL-2 levels were measured in the supernatants of stimulated and unstimulated blood (Figure 2A, right) as previously reported (21). We detected IFN-γ– and/or IL-2–producing, multi-specific anti–SARS-CoV-2 T cell responses (≥2 peptide pools) in 70% (28 of 40) of the individuals from Elgeyo Marakwet and 95% (38 of 40) of the individuals from Kisumu (Figure 2, B and C). Almost all individuals with positive anti-spike serology also showed cytokine responses against multiple SARS-CoV-2 proteins: 94% (15 of 16) of the Elgeyo Marakwet group and 100% (17 of 17) of the Kisumu group. Furthermore, peptide-induced IFN-γ and IL-2 secretion were not only detectable in antibody-seropositive individuals but also in the majority of seronegative ones: Elgeyo Marakwet, 54% (13 of 24) and Kisumu, 91% (21 of 23) (Figure 2, B–D). Thus, only 27.5% of asymptomatic individuals from Elgeyo Marakwet and 5% from Kisumu were negative for both SARS-CoV-2 serology and T cell cytokine analysis (Figure 2D).

Of note, some HIV-infected individuals displayed robust production of IFN-γ and IL-2, and the overall magnitude of SARS-CoV-2–specific T cell responses was not affected by HIV infection (Supplemental Figure 1; supplemental material available online with this article; https://doi.org/10.1172/JCI170011DS1). The HIV status of participants is indicated with an asterisk in Figure 2, B and C; their median CD4^+^ T cell count was 596 cells/μL, with a range of 363–1,376 cells/μL.

**Figure 3:**
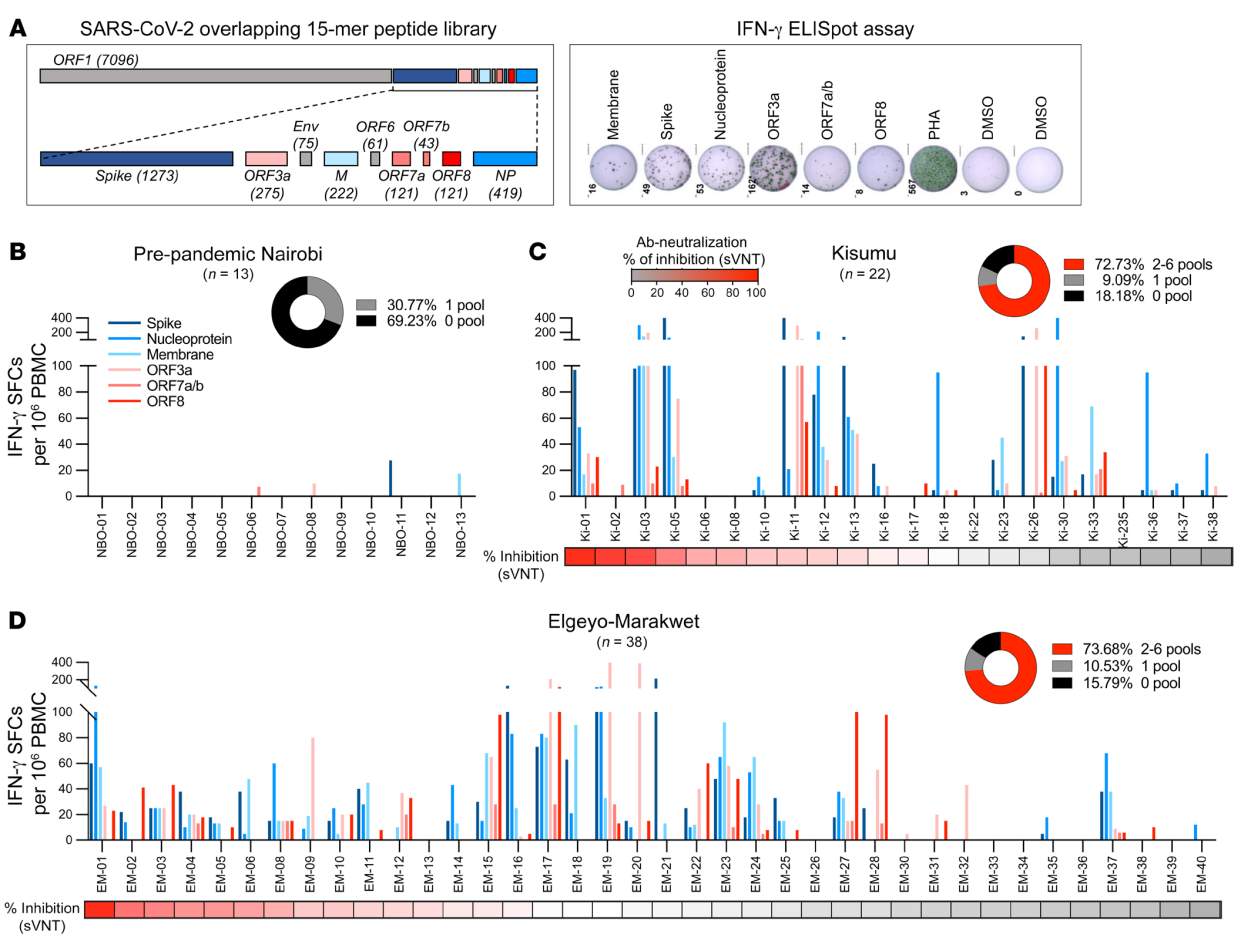
T cells specific for different SARS-CoV-2 proteins in pre-pandemic samples and in the asymptomatic study participants. (**A**) SARS-CoV-2 proteome organization; analyzed proteins are highlighted in color. PBMCs were stimulated with 15 mer peptide pools covering SARS-CoV-2 spike, membrane, and NP structural proteins and ORF3a, ORF7, and ORF8 accessory proteins. IFN-γ–secreting cells (SFCs) in response to peptide stimulation were quantified by ELISPOT assay. The frequency of IFN-γ–secreting cells per 1 million PBMCs is shown for each peptide pool in (**B**) pre-pandemic samples from Nairobi (*n* = 13) and in samples collected in December 2021 from asymptomatic participants from (**C**) Kisumu (*n* = 22) and (**D**) Elgeyo Marakwet (*n* = 38). Participants are organized by level of neutralizing antibodies (percentage of inhibition by sVNT). (**B**–**D**) Pie chart insets show the percentage of participants with responses to 2–6 peptide pools (in red).

The observation that serologically negative, asymptomatic individuals produced T cell cytokines after whole-blood stimulation with peptides covering different SARS-CoV-2 proteins suggests that these individuals possessed virus-specific T cells primed by SARS-CoV-2 infection. Indeed, the simultaneous presence of T cells specific for multiple SARS-CoV-2 proteins is characteristic of previous infection (22). However, since pre-pandemic cross-reactive T cells, usually specific for a single protein, have been observed in 40% to 70% of individuals worldwide (6, 23–27), we performed ELISPOT assays by exposing thawed pre-pandemic PBMCs from Nairobi as well as post-pandemic PBMCs from Kisumu and Elgeyo Marakwet to peptide pools covering the entire lengths of 3 structural (spike, nucleocapsid, membrane) and 3 accessory proteins (ORF3a, ORF7, ORF8) of SARS-CoV-2 (Figure 3A). None of the PBMCs collected before 2019 demonstrated multi-specific T cell responses to SARS-CoV-2 (Figure 3B). Single responses (>5 spots × 10^6^) to a peptide pool covering spike, membrane, ORF3a, or ORF7 were detected in 31% (4 of 13) of the volunteers. In contrast, the ELISPOT assays performed with post-pandemic PBMCs (collected at the same time as the whole blood for T cell assays between November 15 and December 2, 2021) confirmed the presence of T cell reactivity against multiple SARS-CoV-2 proteins in the great majority of the asymptomatic participants from Elgeyo Marakwet and Kisumu (Figure 3, C and D). As the viability of the PBMCs after freezing and thawing was suboptimal (low viability and/or failed positive controls), only 75% (60 of 80) of the samples were analyzable. ELISPOT data confirmed a pattern almost identical to the results obtained using the whole-blood rapid cytokine assays. T cells activated by at least 2 distinct peptide pools were detected in 73% and 74% of PBMCs, respectively, collected in Kisumu and Elgeyo Marakwet. Of note, the results obtained with the whole-blood assay and ELISPOT were identical in the Elgeyo Marakwet group (74% by ELISPOT versus 70% by whole blood), in which the viability of PBMCs was optimal (38 of 40 samples), whereas discrepancies in the frequency of positive responses in the Kisumu group (73% by ELISPOT versus 90% by whole blood) were associated with the poor viability of some samples, implying that the handling of samples can alter T cell immunological results (28).

Finally, to unequivocally demonstrate that peptide pools were activating T cells, selected PBMCs were stimulated with peptides, expanded in vitro, and then analyzed by flow cytometry for the presence of peptide-specific CD4^+^ or CD8^+^ T cells. SARS-CoV-2 peptide–specific CD4^+^ and CD8^+^ T cells were visualized, and their ability to recognize single peptides is shown in Supplemental Figure 2.

**Figure 4:**
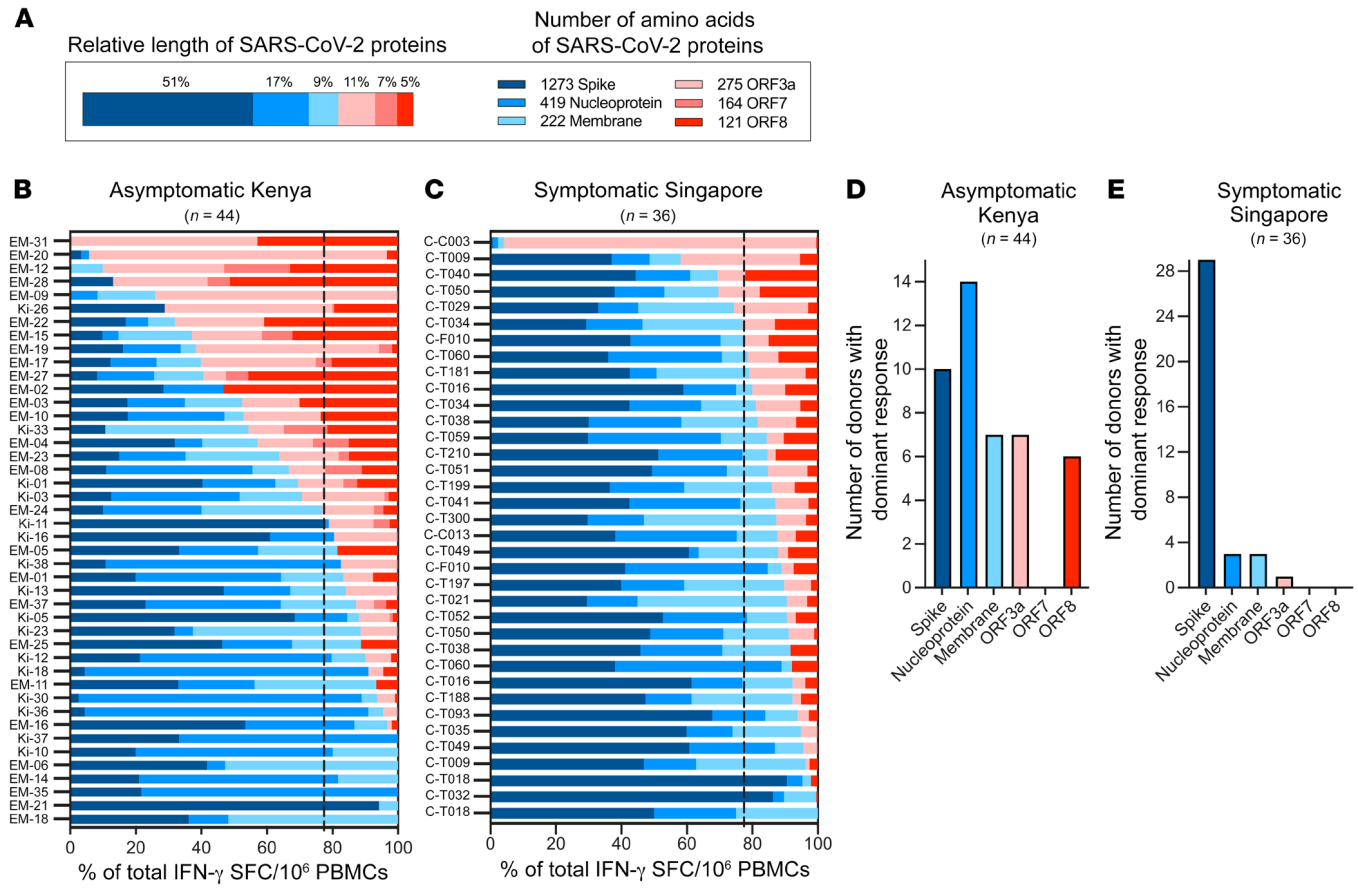
Immunodominance hierarchy of T cell responses to structural and accessory proteins of SARS-CoV-2. (**A**) Schematic representation of the relative length of the 6 different SARS-CoV-2 proteins tested (left) and their number of amino acids (right). (**B**) The SARS-CoV-2 T cell response composition in each responding asymptomatic participant from Kenya (*n* = 44) is shown as a percentage of the total detected response (structural proteins are shown in shades of blue; accessory proteins are shown in shades of red). The dotted line represents the relative length of the structural (77%) and accessory proteins (23%) tested. (**C**) The composition of the SARS-CoV-2 response in convalescent symptomatic COVID-19 patients from Singapore (*n* = 36) is shown as a percentage of the total detected response. The number of participants with a dominant T cell response to the indicated SARS-CoV-2 proteins is shown for samples from asymptomatic participants from Kenya (**D**) and symptomatic convalescent COVID-19 patients from Singapore (**E**).

The utilization of peptide pools covering the whole length of the different structural (spike, membrane, nucleocapsid) and accessory (ORF3a, ORF7, ORF8) proteins in the ELISPOT assays (Figure 4A) permitted the evaluation of the relative T cell immunogenicity of the proteins in 44 asymptomatic Kenyan participants. We calculated the percentage of T cells recognizing each protein in individuals with multi-specific T cells. The bars in Figure 4B show the composition of the SARS-CoV-2 T cell response against different viral proteins for each individual. T cell immunogenicity was not proportional to the length of the protein tested.

For example, despite the fact that spike consisted of 51% of the length of all proteins tested (Figure 4A), anti-spike–specific T cells were the dominant T cell response in only 23% (10 of 44) of tested participants (Figure 4B). Interestingly, we noted a robust T cell response against the accessory proteins ORF3a and ORF8, uniquely present in sarbecoviruses (SARS-CoV-2 and SARS-CoV) (29). Although these proteins represented 15% of the length of all SARS-CoV-2 proteins tested (Figure 4A), ORF3a and ORF8 constituted the dominant response in 30% (13 of 44) of asymptomatic individuals. The T cell immunodominance pattern in asymptomatic individuals living in rural Kenya was, however, distinctive from that in mild-to-moderately symptomatic COVID-19 convalescents (*n* = 36) living in the urban environment of Singapore and tested 6 months after infection with identical methods and peptide pools (Figure 4, C–E). Anti-spike activity clearly represented the dominant T cell response in Singaporean COVID-19 convalescents (Figure 4E). These results are consistent with observations by others who studied COVID-19 convalescents in the United Kingdom and the United States (30–32).

**Figure 5:**
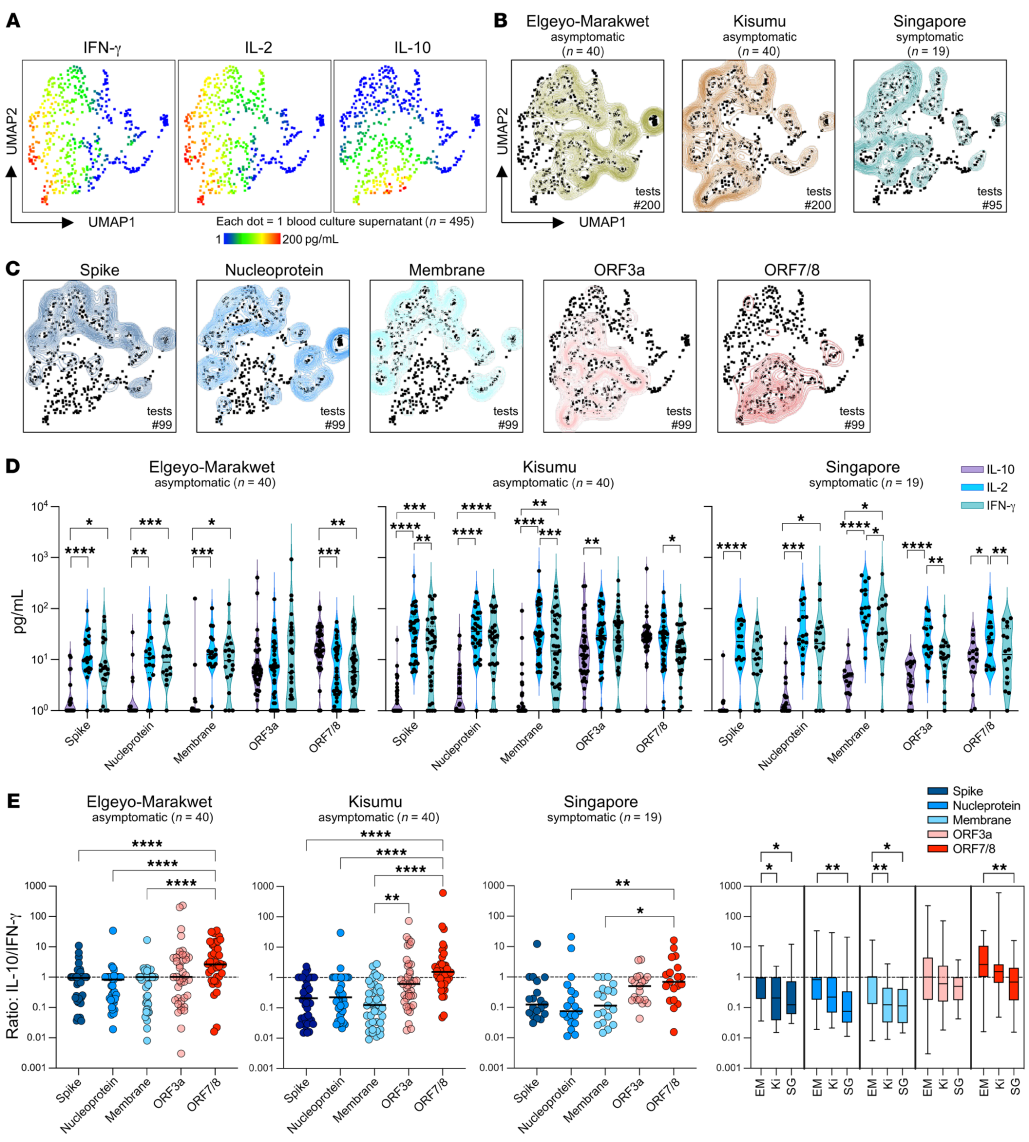
Cytokine secretion profile of SARS-CoV-2 peptide pool–stimulated whole blood from asymptomatic Kenyans and symptomatic convalescent Singaporeans. Whole blood was stimulated with SARS-CoV-2 peptide pools overnight, and the cytokine secretion profile (IFN-γ, IL-2, and IL-10) was analyzed using an unsupervised clustering algorithm UMAP. (**A**) UMAP plots with cytokine secretion heatmaps. (**B**) Concatenated cytokine secretion profiles from asymptomatic participants from Elgeyo Marakwet (left, green, *n* = 40 individuals, *n* = 200 tests) and from Kisumu (middle, brown, *n* = 40 individuals, *n* = 200 tests), and convalescent symptomatic COVID-19 patients from Singapore (right, blue, *n* = 19 individuals, *n* = 95 tests) overlaid on the global UMAP plot of all analyzed samples (black dots; each dot corresponds to 1 culture supernatant). (**C**) UMAP plots comparing the cytokine secretion profiles of whole blood from all individuals tested (*n* = 99) stimulated with the 5 different SARS-CoV-2 peptide pools shown individually. (**D**) Violin plots showing the quantity of IL-10, IL-2, and IFN-γ detected in the different culture supernatants from asymptomatic participants from Elgeyo Marakwet (left) and Kisumu (middle) and from symptomatic convalescents from Singapore (right). Friedman’s test followed by Dunn’s multiple-comparison test (line indicates the median). (**E**) Ratios of IL-10/IFN-γ quantities detected in the culture supernatants stimulated with the different peptide pools. (**F**) Ratios of IL-10/IFN-γ quantities detected in the culture supernatants stimulated with the different peptide pools are compared between the 3 cohorts. EM, Elgeyo Marakwet; Ki, Kisumu; SG, Singapore. (**E** and **F**) Kruskal-Wallis test, followed by Dunn’s multiple-comparison test (line indicates the median). *P < 0.05, **P < 0.01, ***P < 0.001, and ****P < 0.0001.

IL-10 production by virus-specific T cells has been associated with reduced inflammation in respiratory viral infections (33, 34). Therefore, we compared the functional cellular immune response of asymptomatic participants from Kenya with COVID-19 convalescents from Singapore. We used an unsupervised dimension reduction and clustering algorithm (uniform manifold approximation and projection [UMAP]) of the secretomes (IFN-γ, IL-2, and IL-10) of all peptide-stimulated samples (*n* = 495) after subtraction of cytokine levels present in corresponding dimethyl sulfoxide controls. This showed that the secretion of cytokines classically produced by Th1 cells, IFN-γ and IL-2, was overlapping. In contrast, samples with high levels of the regulatory cytokine IL-10 formed a cluster with only partial intersection (Figure 5A). The overall secretomes from the 3 groups of participants differed (Figure 5B). The UMAP from Elgeyo Marakwet displays more samples with no or low levels of cytokine release, consistent with Figure 2 showing that 30% of participants from this group had no SARS-CoV-2–specific cellular immunity. Many secretomes from both asymptomatic groups, Kisumu and Elgeyo Marakwet, clustered with high IL-10 levels, which was not seen for the secretomes from symptomatic COVID-19 convalescents from Singapore. Yet only samples from Kisumu and Singapore clustered on the UMAP with high IFN-γ and IL-2.

Deconvolution of the secretion profiles in response to the individual peptide pools covering the different SARS-CoV-2 proteins showed distinctive profiles between structural and accessory proteins (Figure 5C). Cytokine profiles in response to accessory proteins clustered with high IL-10 secretion. A side-by-side comparison of the cytokines produced by each peptide pool in the 3 cohorts showed that, while structural proteins had a robust Th1 response (high IFN-γ/IL-2, low IL-10), accessory proteins induced similar quantities of IFN-γ and IL-10 (Figure 5D). In the participants from Elgeyo Marakwet, ORF7/8 triggered significantly higher secretion of the antiinflammatory cytokine IL-10 than IFN-γ or IL-2.

The IL-10/IFN-γ ratio was highest for ORF7/8, followed by ORF3a in all 3 groups (Figure 5E). Comparison of the asymptomatic and convalescent groups revealed higher IL-10/IFN-γ ratios in the samples from Elgeyo Marakwet, followed by Kisumu, with the lowest ratios observed in the Singapore group in response to all the tested proteins. The differences in IL-10/IFN-γ ratios in the Elgeyo Marakwet and Kisumu samples were statistically significant only for T cell responses against spike and membrane. However, T cell responses against all the tested proteins, except ORF3a, showed statistically significantly higher IL-10/IFN-γ ratios in asymptomatic participants from Elgeyo Marakwet compared with ratios in the Singaporean COVID-19 convalescents (Figure 5F).

## Discussion

In a unique survey of both cellular and humoral immune responses against SARS-CoV-2 among asymptomatic Africans, we show that 78% of individuals living in 2 rural regions of Kenya, who had no reported respiratory symptoms since December 2019 and who were never knowingly in contact with individuals infected with SARS-CoV-2, possessed broadly reactive T cells specific to multiple SARS-CoV-2 proteins. Sixty percent of these asymptomatic individuals lacked anti-spike antibodies — an antibody response more durable than that of the anti-nucleocapsid antibody (15) — while, among these seronegative participants, 70% had multi-specific T cell responses. The simultaneous detection of a T cell response to different SARS-CoV-2 structural and accessory proteins in these individuals contrasts with the detection of T cell responses specific for single SARS-CoV-2 proteins that we observed in pre-pandemic samples collected in a different geographical area of Kenya. Thus, even though we cannot unequivocally claim that such multi-specific T cell responses demonstrate previous infection by SARS-CoV-2, it is essential to highlight that the cross-reactive T cells found in 30% to 70% of individuals tested before the pandemic (24, 25) were limited to single SARS-CoV-2 proteins (22). In addition, recent work showed that the CD4^+^ T cell response induced by NL-63 and OC-43 coronavirus infections largely do not overlap with SARS-CoV-2–induced CD4^+^ T cells (35). Therefore, we conclude that the detection of T cells specific for different SARS-CoV-2 proteins in the same individual is highly indicative of previous asymptomatic SARS-CoV-2 infection, as shown in seronegative health care workers in the United Kingdom who had been exposed to the virus (22).

Thus, in addition to strengthening the evidence that the rates of asymptomatic SARS-CoV-2 infection were already very high in Kenya before the advent of the highly transmissible Omicron variant of concern, our results strongly suggest that measurement of virus-specific T cells constitutes a far more sensitive assay than the measurement of antibodies to detect past coronavirus infections. This is consistent with the differential waning of antibody titers and T cell frequencies, particularly in individuals with no or minimal symptoms (15) and with the detection of multi-specific SARS-CoV-2 T cell responses in the absence of antibodies in other studies of asymptomatic SARS-CoV-2 infection (11, 12). Furthermore, the persistence of virus-specific T cells over antibodies was also already observed in other coronavirus infections such as SARS-CoV-1 (36) and Middle East respiratory syndrome (MERS) (37, 38). Antibodies against SARS-CoV-1 are undetectable 2 to 3 years after infection (36), while SARS-CoV-1–specific T cells are detectable up to at least 17 years after infection (23). Similarly, T cell responses against MERS coronavirus were present in individuals with occupational exposure to camels in the absence of antibody responses (37, 38).

Despite such evidence, epidemiological assessments of the prevalence of SARS-CoV-2 have mainly utilized serologic assays, since antibodies are easier to measure than T cell responses. However, methodologies for the rapid detection of virus-specific T cells flourished during the COVID-19 pandemic (21, 39–41), including the use of whole blood, which appeared to correlate with protection from SARS-CoV-2 infection in 1 study (41). Here, we show directly that cytokine detection following whole-blood stimulation with peptides can be implemented in locations within a few hours’ distance from a facility with biosafety level-1 cabinets. As these whole-blood assays continue to be perfected for large-scale use, they could eventually be applied routinely for public health surveillance of exposure to microbes that are known to elicit seronegative responses in asymptomatic individuals or in individuals whose antibody levels wane quickly.

Suppose there is indeed a reduced antibody response to SARS-CoV-2, as observed in our cross-sectional study that ended in 2021. In that case, one may ask why there has been a steady increase in the seroprevalence of SARS-CoV-2 in Kenya and other countries of the African continent, reaching 90% or higher in many settings? Certainly, our study targeted asymptomatic individuals known to have reduced antibody positivity, whereas serosurveys encompass both symptomatic and asymptomatic persons. Nevertheless, there remains a wide discrepancy between the antibody and T cell responses. A clue comes from data in surveillance platforms in Kenya, where a 5% to 10% PCR positivity for SARS-CoV-2 has been observed among symptomatic individuals during interwave periods. We speculate that as SARS-CoV-2 transitioned from an epidemic to an endemic virus, continual reinfections have boosted the population’s antibody levels, especially as there has been very little promotion of nonpharmaceutical interventions in over a year. The fact that our participants were from a rural area and had at least some degree of isolation from urban centers may have allowed time for their antibody levels to decline rather than get boosted through reinfection.

We observed that the Kisumu group of asymptomatic participants had a 95% multi-specific T cell response to SARS-CoV-2 proteins, while the proportion was lower (70%) for participants from Elgeyo Marakwet. We attribute this difference to the greater interaction of residents of peri-urban Kisumu with the city of Kisumu, which boasts a population of approximately 350,000, whereas the residents of Elgeyo Marakwet whom we enrolled live a life distinctly more remote from urban centers.

These 2 rural communities have health and environmental characteristics that one may speculate influence the unique immune responses we observed. In both communities, administering a birth dose of BCG is routine, incident *Mycobacterium tuberculosis* (TB) disease, and helminthic infections are commonplace (school children regularly receive anti-helminthics during biannual mass drug administration events, and persons living with HIV are routinely provided TB-preventive therapy). Although malaria is uncommon in Elgeyo Marakwet, it is endemic in Kisumu. Furthermore, while both groups often sleep in the same shelter as their livestock to prevent their theft, those in Elgeyo Marakwet typically own cows, prepare a fermented cow’s milk called mursik, and exercise more because of the steep terrain that approximately 1,400 meters higher in altitude than Kisumu. Whether these environmental differences explain some of the unexpected features of SARS-CoV-2 T cell responses observed in asymptomatic rural Kenyans will require additional investigation.

We observed 2 distinctive immunologic characteristics among these asymptomatic rural Africans. The first was the observation of an unexpectedly strong T cell immunogenicity against the viral accessory proteins ORF3a and ORF8 that contrasted with the SARS-CoV-2 T cells studied in convalescent urban Singaporeans and Western (United Kingdom and United States) patients, who instead showed a clear dominance of spike-specific T cell responses (30–32). Of note, ORF3a and ORF8 proteins are unique to SARS-CoV-2 and SARS-CoV (sarbecovirus), but are not present in common seasonal coronaviruses (29). Hence, we can exclude the notion that this peculiar T cell dominance is caused by cross-reactive memory T cells induced by seasonal coronaviruses. However, exposure to commensal antigens can modulate the SARS-CoV-2 T cell repertoire (6). Thus, the possibility that differing microbiomes can alter the SARS-CoV-2 T cell immunodominance cannot be excluded. An alternative hypothesis emerges from the kinetics of SARS-CoV-2 protein synthesis. Accessory molecules (ORF3a and ORF7a/b) are produced earlier after infection (42), probably because they play a role in suppressing innate immunity in the infected cells (43). Furthermore, ORF3a, ORF7a/b (44), and ORF8 (45) have been shown to reduce HLA class I presentation. The hypothesis is that, in the early phases of infection, these proteins might be more immunogenic than structural proteins, and in asymptomatic infections, such abortive infections with limited virion production may predominate as compared with symptomatic infections. Support for this hypothesis is the finding of an early immune response against accessory proteins in patients with acute COVID-19 (46).

The second intriguing feature was the skewed IL-10 production by SARS-CoV-2 T cells detected preferentially in asymptomatic individuals living in rural Kenya, particularly in the rural communities of Elgeyo Marakwet. While genetic factors might be the basis of such a difference, another interpretation is that specific environmental factors more common in rural areas of Kenya than in very urbanized Singapore altered the cytokine profile of SARS-CoV-2 T cells. The ability of helminths or mycobacteria (including BCG) to skew the cytokine production of immune cells has been well documented (47, 48), but other factors may also play a role. For example, compared with Kisumu participants, the relatively augmented levels of IL-10 production observed in participants from Elgeyo Marakwet could be explained by the differences in lifestyle and diet or by the absence of malaria in this high-altitude region. Exercise (49) and fermented foods (50) are associated with antiinflammatory effects on the human immune response, whereas malaria has been shown to induce a Th1-like response (51). It is worth pointing out that high levels of IL-10 were also detected in a study of asymptomatic infection occurring in Singapore at the beginning of the COVID-19 pandemic. In this case, the asymptomatic individuals were all young guest laborers who had recently arrived from Bangladesh (12). Whether the observed ability of SARS-CoV-2 T cells to produce more IL-10 by can fully explain the asymptomatic profile of SARS-CoV-2 infection observed in these groups deserves further study.

Our study has several limitations. First, the study participants were not selected at random from local communities but rather were targeted by community health care workers because of the likelihood of the individuals’ uninfected status and their minimal contact with major urban centers. The small number of participants from these communities limits the generalizability of our findings to the rest of Kenya or perhaps even within these communities. Our comparison group for symptomatic convalescent individuals consisted of hospitalized Singaporean patients with mild-to-moderate COVID-19 and nonhospitalized individuals from these same communities. Finally, as already highlighted, our pre-pandemic PBMCs were from a healthy Nairobi cohort and not from individuals from either Kisumu or Elgeyo Marakwet because pre-pandemic PBMCs from healthy volunteers in these counties were unavailable.

Our finding that cellular immune assays are more sensitive than antibody assays in detecting SARS-CoV-2 infection in an African population in which asymptomatic infections have predominated implies that seroprevalence surveys might underestimate the spread of COVID-19 in Africa (52). This observation, coupled with the knowledge that coronaviruses have the propensity to evolve into diseases with pandemic potential, should spur on the development of simple and scalable cellular immune assays to test populations for public health purposes. To determine whether our findings are generalizable, we encourage the public health research community to conduct similar studies on a much wider scale. We also observed that the T cell responses of asymptomatic residents of these rural Kenyan communities were unusually directed against SARS-CoV-2’s nonstructural proteins and were skewed toward an antiinflammatory response. These data support the call for a more in-depth analysis of the impact of environmental factors on the development of protective or pathological antiviral immunity against SARS-CoV-2 to understand COVID-19 pathogenesis not only in Africa but around the world.

## Methods

### Study design

We worked with local health departments and 1 facility each in Kisumu and Elgeyo Marakwet to identify a convenience sample of 40 potential volunteers who lived in rural parts of each county. Participants enrolled in this cross-sectional study had to be at least 18 years of age and must not have received a COVID-19 vaccine. In addition, because the enrollees were intended to be individuals not exposed to SARS-CoV-2, they must have had no history of respiratory illness (cough, shortness of breath, fever, or sinus congestion) since December 31, 2019; no history of travel to or meeting with persons from high-incidence counties such as Nairobi or Mombasa (i.e., having >500 cumulative cases per 100,000 as of January 2021); no history of incarceration since January 2020; and a negative SARS-CoV-2 PCR or antigen test at the time of enrollment. Kibigori and Tamu in Kisumu county are a 1.5-hour motorbike and bus ride away from Kisumu city; residents are engaged in subsistence agriculture and seasonal work on sugar plantations but could socialize with visiting city dwellers at local venues. Residents of Chesoi, Kapcherop, and Tambach in Elgeyo Marakwet are also engaged in subsistence farming but infrequently interact with dwellers of the nearest large city, Eldoret, a 2-hour bus ride away and located in a different county.

We enrolled participants in Kisumu between November 15 and November 18, 2021, and in Elgeyo Marakwet between November 29 and December 2, 2021. Up to 18.2 mL of blood was drawn from each volunteer and transported under controlled temperature, and a nasopharyngeal swab was collected to perform a PCR test to detect the presence of SARS-CoV-2. Additional consent was received to conduct an HIV rapid antibody test and, if accepted, to provide HIV prevention counseling.

As pre-pandemic frozen PBMCs from either Kisumu or Elgeyo Marakwet were unavailable, we tested cross-reactivity against SARS-CoV-2 proteins in frozen PBMCs collected from healthy volunteers in Nairobi between October 19, 2015, and July 19, 2016. These samples came from the Simulated Vaccine Efficacy Trial (SiVET) (53).

### Laboratory procedures

#### Antibody assays.

SARS-CoV-2–neutralizing antibodies were analyzed using a surrogate neutralization assay (cPass, GenScript Biotech) that measures how neutralizing antibodies in the serum bind to the HRP-labeled SARS-CoV-2 RBD and prevent it from binding to the hACE2 protein (19). RBDs from 3 different SARS-CoV-2 variants were tested: the ancestral Wuhan strain, the Delta variant, and the Omicron variant. A threshold of 30% inhibition was considered a positive result. Additionally, a 2-step antibody detection test was conducted using the SCoV-2 Detect IgG ELISA (InBios) as the initial test, followed by the EUROIMMUN anti–SARS-CoV-2 ELISA as a confirmatory test for participants who were positive by the InBios test. NP-specific antibodies were tested with the anti–SARS-CoV-2 NCP ELISA (IgG) test (EUROIMMUN).

#### Cytokine release assays using whole blood.

Details of this method are published elsewhere (21). Freshly drawn whole blood (320 μL) was stimulated with 5 distinct 15 mer peptide pools and controls (Figure 2A). After overnight culturing at 37°C with 5% CO_2_, the supernatant (plasma) was collected and frozen at –80°C for later shipment to our Singapore laboratory. Cytokine concentrations in the plasma were quantified using an Ella machine measuring IFN-γ, IL-2, and IL-10, following the manufacturer’s instructions (ProteinSimple). The level of cytokines present in the plasma of DMSO controls was subtracted from the corresponding levels in the peptide pool–stimulated samples. Samples with cytokine quantities (IFN-γ, IL-2, IL-10) above to 5 pg/mL were considered positive. Since SARS-CoV-2 infection usually induces T cells specific not for single but multiple SARS-CoV-2 proteins (22), we scored as “T cell–positive” only the individuals who had a positive response to at least 2 peptide pools for each cytokine tested or a “multi-specific” response.

Subsequently, concentrations of each cytokine in all culture supernatants were transformed using the logical transformation function, and UMAP was run using a 15 nearest neighbors (nn), min_dist of 0.5 and Euclidean distance (54). The results obtained from UMAP analyses were incorporated as additional parameters and converted to FCS files, which were then loaded into FlowJo (BD Biosciences) to generate heatmaps of cytokine secretion on the reduced dimensions.

#### SARS-CoV-2 peptide–specific T cell quantification by ELISPOT.

The frequency of SARS-CoV-2 peptide–specific T cells was quantified as described previously (43). Briefly, cryopreserved PBMCs that had been shipped to Singapore were thawed and stimulated with the following 15 mer peptide pools overlapping by 10 amino acids in ELISPOT plates: structural (NP, membrane, spike) and accessory (ORF3a, ORF7, ORF8). The plates were then incubated with a human biotinylated IFN-γ detection antibody, followed by streptavidin–alkaline phosphatase (streptavidin-AP) and developed using the KPL BCIP/NBT phosphatase substrate (Seracare Life Sciences). The results are expressed as spot-forming cells (SFCs) per 10^6^ PBMCs.

#### Cell culture for T cell expansion.

T cell lines were generated as follows: 20% of the PBMCs were pulsed with 10 μg/mL overlapping SARS-CoV-2 peptides for 1 hour at 37°C and then washed and cocultured with the remaining cells in AIM-V medium (Gibco, Thermo Fisher Scientific) supplemented with 2% AB human serum (Gibco, Thermo Fisher Scientific). T cell lines were cultured for 10 days in the presence of 20 U/mL recombinant IL-2 (R&D Systems).

#### Flow cytometry.

PBMCs were stimulated with peptide pools and expanded in vitro for 10 days as described before (23). Expanded T cell lines were stimulated for 5 hours at 37°C with or without SARS-CoV-2 peptide pools (2 μg/mL). After 1 hour, 10 μg/mL brefeldin A (MilliporeSigma) and 1× monensin (BioLegend) were added. Cells were stained with the yellow LIVE/DEAD fixable dead cell staining kit (Invitrogen, Thermo Fisher Scientific) and the surface markers anti-CD3 (SK7 or OKT3; BioLegend), anti-CD4 (SK3, BD Biosciences), and anti-CD8 (SK1, BD Biosciences). Cells were subsequently fixed and permeabilized using the Cytofix/Cytoperm kit (BD Biosciences) and stained with anti–IFN-γ (25723; R&D Systems) and anti–TNF-α (MAb11, BD Biosciences) antibodies and analyzed on a CytoFLEX (Beckman Coulter). Data were analyzed by FlowJo (BD Biosciences).

### Statistics

Quantities of IL-10, IL-2, and IFN-γ detected in the different culture supernatants from asymptomatic participants from Elgeyo Marakwet and Kisumu and from symptomatic convalescents from Singapore were compared using the Friedman test followed by Dunn’s multiple-comparison test (Figure 5D). Ratios of IL-10/IFN-γ quantities between different peptide pools (Figure 5E) and between the 3 cohorts (Figure 5F) were analyzed with the Kruskal-Wallis test, followed by Dunn’s multiple-comparison test. All lines indicate the median. In Figure 5E, whiskers represent the minimum and maximum values, and the box indicates the lower and upper quartiles.

### Study approval

Written informed consent was received from all participants. The Scientific and Ethics Review Unit of the KEMRI approved the protocol (KEMRI/RES/7/3/1, protocol no. 4186). The Kenya National Commission for Science, Technology and Innovation provided a permit (license no. NACOSTI/P/21/12171), and the US Centers for Disease Control and Prevention relied on the KEMRI approval (CDC no. 7353) as did the Washington State University IRB. SiVET received ethics approval from the Kenyatta National Hospital/University of Nairobi Ethics and Research Committee (P137/03/2015). Blood was also collected 6 months after SARS-CoV-2 infection from convalescent Singaporeans who had symptomatic COVID-19 in 2020 under a COVID-19 PROTECT study group protocol, which was approved by the National Healthcare Group (NHG) Domain Specific Review Board (DSRB) (nos. 2012/00917 and NHG DSRB E 2020/00091). All participants provided written informed consent in accordance with the Declaration of Helsinki for Human Research.

### Data availability

All data are available upon request.

## Author contributions

TS, JBO, AB, and NLB conceptualized the study and designed the experiments. JBO, PAM, AM, JO, GMM, RKB, CO, PM, NT, SKH, and SH recruited participants, collected samples, and performed the experiments. NLB, AB, and TS analyzed the data. NLB, TS, and CAD prepared the figures and the table. AB, TS, AHR, and DCL acquired funding for the project. AB, TS, EO, NLB, KM, OA, FOO, and AHR were involved in project administration. The study was supervised by KM, CO, GMM, OA, AB, and NLB. TS and AB wrote the original draft of the manuscript, which was reviewed and edited by AB, TS, NLB, EO, JO, RKB, CO, and DCL.

## Supplementary Material

Supplemental data

ICMJE disclosure forms

## Figures and Tables

**Table 1:**
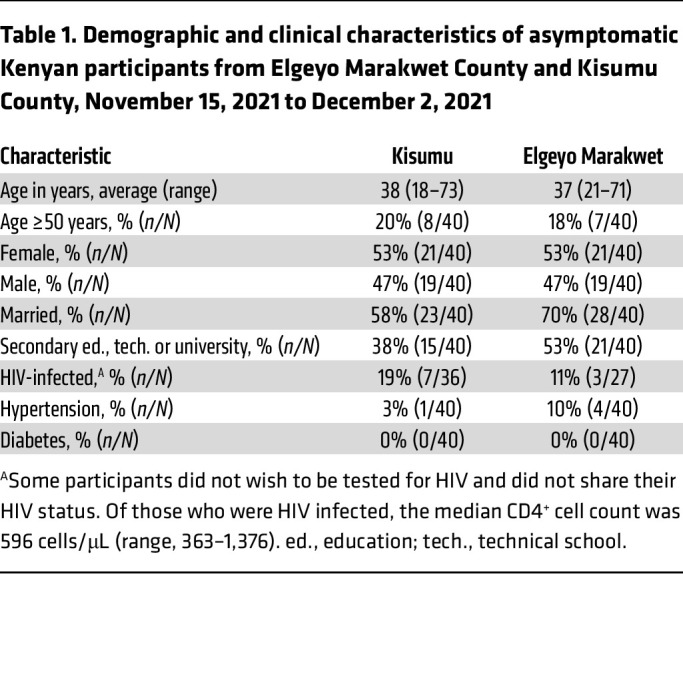
Demographic and clinical characteristics of asymptomatic Kenyan participants from Elgeyo Marakwet County and Kisumu County, November 15, 2021 to December 2, 2021

## Version 1 05/23/2023

In-Press Preview

## Version 2 07/03/2023

Electronic publication
