# Supplementary material for: Prevalence and functional profile of SARS-CoV-2 T cells in asymptomatic Kenyan adults
Source: J Clin Invest. 2023 Jul 3;133(13):e170011. doi: 10.1172/JCI170011 (PMC10313370; doi:10.1172/JCI170011)
Supplement: Supplemental data [file jci-133-170011-s126.pdf]

Supplementary Figures

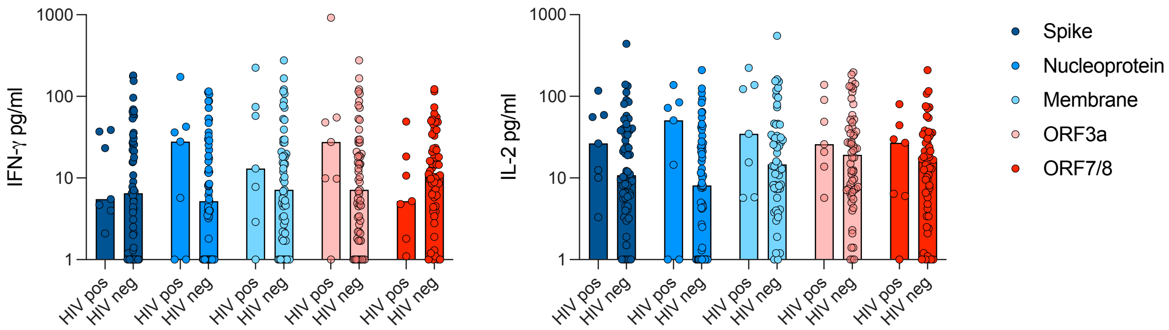

Figure S1. Similar level of SARS-CoV-2-peptide pool induced cytokine release in HIV infected participants

IFN-γ and IL-2, secreted in response to SARS-CoV-2 peptide-pool stimulation, were quantified in the plasma of the blood cultures. If peptide stimulation induced >5pg/ml of cytokines (IFN-γ and/or IL-2) above corresponding DMSO controls with two distinct peptide pools, the individual was considered positive for SARS-CoV-2-specific T cells. IFN-γ and IL-2 concentrations from individuals considered positive for SARS-CoV-2-specific T cells with positive and negative HIV status are shown side-by-side.

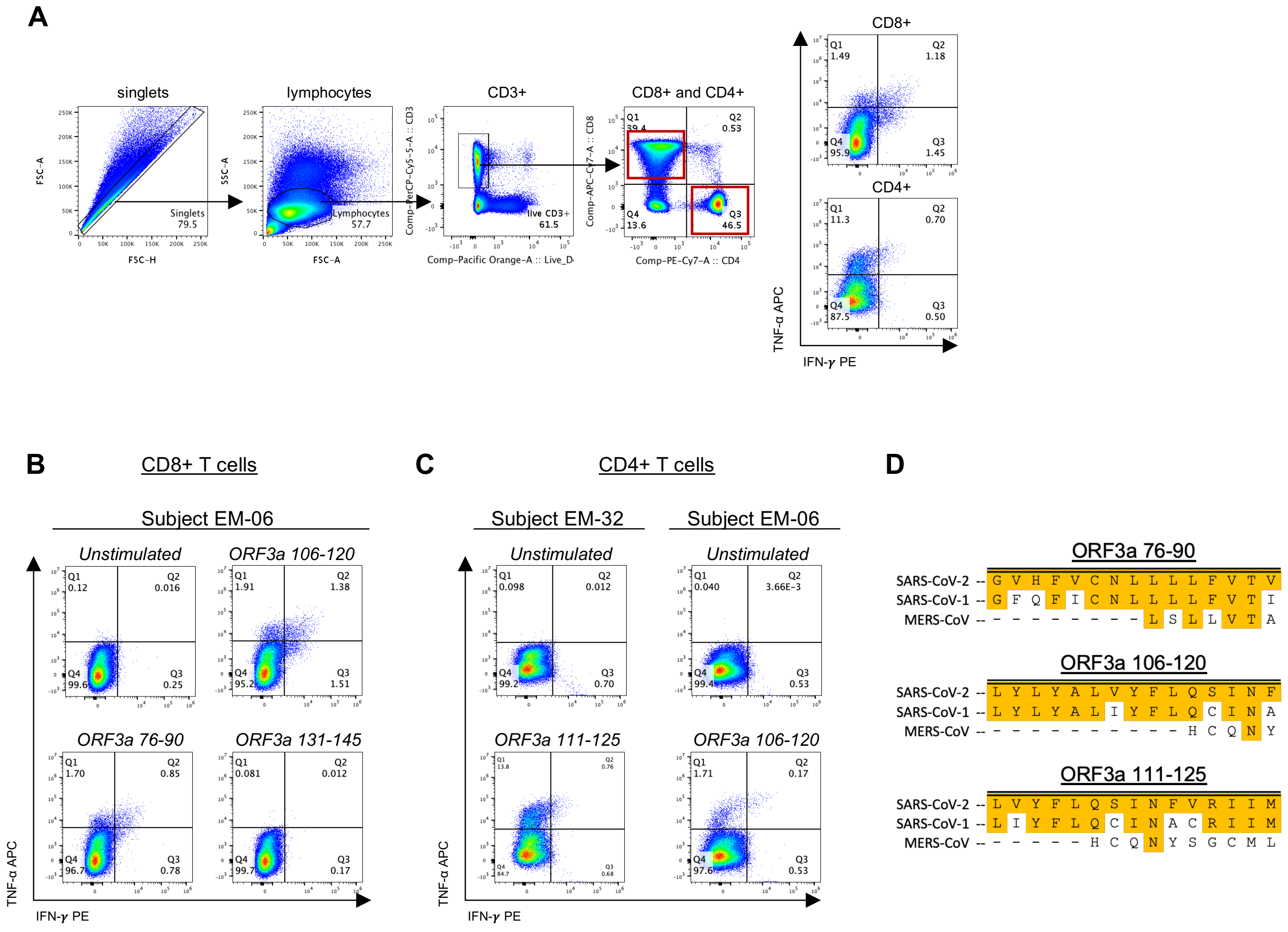

Figure S2. Phenotypic analysis of T cells specific to single SARS-CoV-2 peptides

PBMC were in vitro expanded with peptide pool ORF3a for 10 days and responses to individual peptides were identified stimulating the expanded cells with a peptide matrix strategy using an ELISpot assay. Responses to single peptides were then confirmed by flow cytometry analysis. (**A**) Flow cytometry gating strategy. (**B**) Participant EM-06 with CD8 T cell responses specific to two different peptides within ORF3a. (**C**) Participants EM-32 and EM-06 with CD4 T cell responses specific to two peptides within ORF3a. (**D**) The sequences of identified T cell epitopes that were confirmed by ICS are aligned with the corresponding sequence of SARS-CoV-1 and MERS-CoV, the only other coronaviruses known to infect humans that expresses ORF3a.
